# ROS-mediated up-regulation of SAE1 by *Helicobacter pylori* promotes human gastric tumor genesis and progression

**DOI:** 10.1186/s12967-024-04913-5

**Published:** 2024-02-13

**Authors:** Liu Shi, Jianfang Shangguan, Ying Lu, Jianfang Rong, Qinyu Yang, Yihan Yang, Chuan Xie, Xu Shu

**Affiliations:** 1 Department of Gastroenterology, The First Affiliated Hospital of Nanchang University, NO. 17 Yongwaizheng Street, Nanchang, 330006 Jiangxi China; 2 Department of Gastroenterology, The Affiliated Ganzhou Hospital of Nanchang University, No.16, Meiguan Avenue, Ganzhou, 341000 Jiangxi China

**Keywords:** SAE1, SUMOylation, Gastric cancer, *Helicobacter pylori*, ROS, EMT

## Abstract

Helicobacter pylori (*H. pylori*) is a major risk factor of gastric cancer (GC). The SUMO-activating enzyme SAE1(SUMO-activating enzyme subunit 1), which is indispensable for protein SUMOylation, involves in human tumorigenesis. In this study, we used the TIMER and TCGA database to explore the SAE1 expression in GC and normal tissues and Kaplan–Meier Plotter platform for survival analysis of GC patients. GC tissue microarray and gastric samples from patients who underwent endoscopic treatment were employed to detect the SAE1expression. Our results showed that SAE1 was overexpressed in GC tissues and higher SAE1 expression was associated with worse clinical characteristics of GC patients. Cell and animal models showed that *H. pylori* infection upregulated SAE1, SUMO1, and SUMO2/3 protein expression. Functional assays suggested that suppression of SAE1 attenuated epithelial-mesenchymal transition (EMT) biomarkers and cell proliferation abilities induced by *H. pylori*. Cell and animal models of ROS inhibition in *H. pylori* showed that ROS could mediate the *H. pylori*-induced upregulation of SAE1, SUMO1, and SUMO2/3 protein. RNA sequencing was performed and suggested that knockdown of SAE1 could exert an impact on IGF-1 expression. General, increased SUMOylation modification is involved in *H. pylori*-induced GC.

**Supplementary Information:**

The online version contains supplementary material available at 10.1186/s12967-024-04913-5.

## Background

Gastric cancer (GC) is listed as the fifth most frequently diagnosed cancer and the fourth leading cause of cancer-related mortality globally [1]. *Helicobacter pylori* (*H. pylori*) is classified as a type-I human carcinogen. It initiates a chronic inflammatory response in the gastric mucosa, contributing to the occurrence of gastritis that can trigger a multistep gastric tumorigenesis cascade [2]. The chronic proinflammatory condition leads to the emergence of excessive reactive oxygen species (ROS) generation, resulting in oxidative DNA damage and the activation of oncogenic signaling pathways [3, 4]. During durative infection with *H. pylori*, the cytotoxin-associated gene A (CagA) protein is involved in the multiple “hits” on the gastric mucosa [5, 6]. Generally, clarifying the mechanisms of *H. pylori*-induced gastric carcinogenesis is valuable to the clinical treatment of *H. pylori* infection and GC.

SUMOylation, a posttranslational modification that attaches the addition of small ubiquitin-like modifier (SUMO) molecules to an acceptor lysine of target proteins, is performed by three enzymatic cascade steps [7]. SUMOylation is an essential mechanism in cellular reactions to various stresses [8]. ROS have been reported to affect SUMOylation and deSUMOylation homeostasis [9]. Enzymes relevant to SUMOylation are dysfunctional in many human pathological processes [10], which disturbs the balance of SUMO-modified cellular substrates, eventually resulting in tumorigenesis [11, 12]. SAE1 (SUMO-activating enzyme subunit 1) is an essential heterodimeric SUMO-activating effector. It can regulate the activation of ATP-dependent SUMO protein by connecting SUMO protein and SAE2 dependent on a thioester bond, thus participating in the modification process of SUMO protein [13]. Upregulated expression of SAE1 was reported in the progression of human cancers, including glioma [14], and hepatocellular carcinoma (HCC) [15]. Currently, the biological effect of SAE1 in *H. pylori* infection-related GC is still underexplored.

The biological program termed epithelial-to-mesenchymal transition (EMT) is heralded as a key hallmark of carcinogenesis [16]. EMT is actuated by a series of transcription factors including, leading to the upregulation of mesenchymal markers and suppression of epithelial markers [17]. Previous studies have shown that *H. pylori* promotes the EMT program in GC dependent on CagA [18]. Additionally, it has been experimentally documented that SUMOylation of several transcription factors can regulate EMT program [19].

Our study explored the effect of SAE1 on *H. pylori*-induced carcinogenesis. We searched for the SAE1 expression in the cancer database and further verified it with a tissue microarray. Cell and animal models were utilized to investigate the association among SAE1, SUMO1, SUMO2/3, ROS, and *H. pylori*-associated GC. RNA sequencing was performed to identify potential mechanisms of SAE1 in GC.

## Methods

### Bioinformatics analysis

The gene expression levels of SAE1 in some cancers, including GC were analyzed by the Tumor Immune Estimation Resource (TIMER) database (https://cistrome.shinyapps.io/timer/,TIMER 2.0). SAE1 expression in GC and the clinical information of GC patients were derived from TCGA database (https://portal.gdc.cancer.gov/,version 39.0, December 4, 2023). The expression difference of SAE1 between GC tissues and adjacent tissues was analyzed and imaged using R3.6.3 software. We searched for the SAE1 gene using the Kaplan–Meier Plotter data analysis platform [20] (http://kmplot.com/analysis/) for survival analysis of GC patients.

### Human gastric specimens and tissue microarray

We collected a total of 80 human gastric specimens extracted from individuals who received endoscopy treatment at the First Affiliated Hospital of Nanchang University. The research protocol and the exemption informed consent were approved by the Ethics Committee of the First Affiliated Hospital of Nanchang University. These samples consisted of 40 cases of chronic non-atrophic gastritis (CNAG) and 40 cases of intestinal metaplasia (IM). The samples were divided into *H. pylori*-infected subgroup and *H. pylori*-uninfected subgroup. A GC tissue microarray slide (HStmA180Su19, Outdo Biotech, Shanghai) including 93 tumor tissues with 93 paired adjacent tissues was purchased.

### Animal models

Animal care and experimental protocols were performed in accordance with the guidelines established by the Institutional Animal Care and Use Committee of Nanchang University. Animal models including uninfected control, *H. pylori* strain PMSS1(Cag A^+^), and *H. pylori* strain PMSS1(Cag A^−^) group were performed on thirty 6- to 8-week-old male C57BL\6 mice. The three groups were administered 1 ml orogastric infusions of sterile Brucella broth, 1 ml orogastric infusions of 1 × 10^9^ colony-forming units of *H. pylori* strain PMSS1(Cag A^+^), and 1 ml orogastric infusions of 1 × 10^9^ colony-forming units of *H. pylori* strain PMSS1(Cag A^−^), respectively, once every other day for a total of ten infusions. Each group was sacrificed at 3 months after initial *H. pylori* infection. Protein was extracted from mouse gastric tissue for Western blot analyses.

Another animal model, including the uninfected group, *H. pylori* infection group, and *H. pylori* infection + NAC treatment group, was established as previously described [21]. Briefly, thirty-six 6- to 8-week-old clean-grade male Balb/c mice were divided into three groups (12 mice per group). Group one was given orogastric infusions of sterile Brucella broth as an uninfected control. Group two was given orogastric infusions of *H. pylori*-type strain SS1 (Cag A +). Group three was given orogastric infusions of *H. pylori* strain SS1(Cag A +) followed by NAC treatment. The samples were embedded in paraffin for storage after being fixed in 4% paraformaldehyde. The blocks were then cut into 5-μm-thick sections and fixed on glass slides for immunohistochemistry (IHC) staining. All institutional and national guidelines for the care and use of laboratory animals were followed.

### Immunohistochemical analysis.

IHC staining was conducted to detect SAE1 expression in the tissue microarray, human gastric specimens and gastric samples of Balb/c mice. Two pathologists who were blinded to detailed information of tissue sections were in charge of scoring the degree of IHC staining separately. The IHC score was computed by multiplying the staining intensity score (levels 0, 1, 2 or 3 indicated negative, weakly positive, moderately positive or strongly positive, respectively) by the frequency score (levels 0, 1, 2, 3 or 4 represented positive areas of 0–5%, 6–25%, 26–50%, 51–75% or 76–100%, respectively).

### Cell culture and reagents

The Human gastric cell lines GES-1 and AGS were cultured in RPMI 1640 (Gibco, Invitrogen, USA) medium supplemented with 10% fetal bovine serum (FBS) and 1% penicillin/streptomycin (Gibco, Invitrogen, USA). Cells were incubated in humidified air containing 5% CO2 at 37 °C. The culture medium was replaced every other day.

According to specific experimental requirements, cells were treated with the ROS inhibitor N-acetylcysteine (NAC, Sigma, USA) or H_2_O_2_ (30%, Wei Ao, China). H_2_O_2_ was diluted with pure water.

### siRNA transfection

Small interfering RNAs (siRNAs) directed against SAE1 and negative control siRNAs (Si-NC) were purchased from Gene Pharma (Shanghai, China). Transient transfection was conducted according to a standard protocol provided by the manufacturer. After transfection with siRNA for 48–72 h, the cells were utilized for western blot analysis and functional study. The siRNA sequences targeting SAE1 were listed in the Additional file 1: Table S1.

### *H. pylori* strains

*H. pylori* strain PMSS1(Cag A^+^), *H. pylori* strain PMSS1(Cag A^−^), and *H. pylori* strain SS1 (Cag A^+^) were used in this study. All *H. pylori* strains were cultured on Campylobacter agar plates with 10% sheep serum at 37 °C under microaerophilic conditions. Gastric cells were cocultured with *H. pylori* strains at different multiplicities of infection (MOI) or for different times when they reached 70% confluence.

### Western blot analysis

AGS and GES-1 cells were treated with a mixture of RIPA lysis buffer (Beyotime, Shanghai, China) and a protein phosphatase inhibitor (Sigma, USA) for 30 min on ice. After detection of total protein concentration via a BCA assay kit (Thermo Scientific, GA, USA), equal amounts of protein were separated by 10% SDS polyacrylamide gel and transferred to nitrocellulose membranes, which were blocked with 5% nonfat milk at room temperature for 1 h and then incubated with specific primary antibodies at 4 °C overnight. The membranes were washed three times with Tris-buffered saline supplemented with 0.1% Tween (TBST) (Solarbio Biotechnology) and then incubated with a horseradish peroxidase-conjugated secondary antibody (Beyotime, Shanghai, China) at room temperature for 1 h, followed by an enhanced chemiluminescence kit (Thermo Fisher Scientific, GA, USA). β-actin served as an internal control. Protein abundances were detected by an iBright imaging system (Thermo Fisher Scientific, GA, USA). The specific primary antibodies used were listed in the Additional file 1: Table S2.

### Cell proliferation assay

Proliferation was detected by Cell Counting Kit-8 (CCK8, Trans Gen Biotech, China) according to the manufacturer’s protocol. The treated cells were resuspended and seeded in 96-well plates at a concentration of 2 × 10^3^ cells per well and incubated in the previously described environment for 24, 48, and 72 h. Each well was treated with 100 μl culture medium containing 10 μl CCK- 8 solution and cultured for 1 h. The absorbance was detected at a wavelength of 450 nm by a Molecular Devices Spectra Max M2e.

### Colony formation assay

The treated cells were resuspended and seeded in 6-well plates at an initial concentration of 300 cells/well. The cells were incubated for up to 10–14 days. After being washed twice with phosphate buffered saline (PBS), the colonies were fixed in 4% paraformaldehyde (Solarbio, P1110, Beijing, China) for 15 min at room temperature, stained with 0.1% crystal violet solution (Solarbio, G1046, Beijing, China) for 15 min at room temperature and then washed again with double-distilled H_2_O. Images of the colonies were photographed and the colony forming efficiency was calculated.

### Transwell assay

A 24-well plate chamber insert (BD Biosciences, USA) was utilized for Transwell assays. For the migration assay, 4 × 10^4^ cells per well resuspended in 400 μl serum-free medium were seeded in the upper chamber insert, and the lower chamber was filled with 600 μl medium containing 20% FBS. After 48 h of incubation, the cells were washed twice with PBS, fixed with 4% paraformaldehyde for 15 min, and stained with 0.5% crystal violet solution for 15 min. The cells remaining on the upper surface of the chamber insert were carefully removed by a cotton ball. The positively stained cells were counted under a microscope. For the invasion assay, Matrigel-precoated inserts (BD, Biosciences, USA) were used at the bottom of the chamber inserts used in the migration assay.

### Wound-healing assay

A total of 10^6^ treated cells were plated on a six-well plate. Scratch wounds were carefully generated using a sterile pipette tip when cells reached more than 90% confluence, and the cellular debris was washed by PBS. Pictures were captured at 0 and 24 h after wounding.

### ROS detection

The ROS level of treated cells was measured by Reactive Oxygen Species Assay Kit (Yeasen Biotechnology, Shanghai, China). 96-well plates were seeded with resuspended cells at a density of 10^4^ cells/well. After cocultivation with PMSS1 strain for 12 h, 10 mmol/L NAC for 12 h, combination of PMSS1 strain and 10 mmol/L NAC for 12 h, and 200 μmol/L H_2_O_2_ for 1 h, the cells were washed with PBS and cultivated with DCFH-DA in the dark for 30 min. After an additional washing with PBS, the level of ROS in the cells was imaged and analyzed by a high-content fluorescence microscope (IN Cell Analyzer2200, GE, USA).

### RNA-sequencing analysis

Total RNA was extracted using TRIzol reagent (Invitrogen, GA, USA). The NanoDrop2000 spectrophotometer (Thermo Scientific, USA) was utilized to RNA purity and quantification evaluation. RNA integrity was assessed using the Agilent 2100 Bioanalyzer (Agilent Technologies). Then, the construction of RNA-sequence libraries was based on the protocol of the TruSeq Stranded mRNA LT Sample Prep Kit (Illumina). Genes were filtered based on the mean counts, and only genes with a mean count greater than 2 were selected for further analysis. The DESeq2 software was used to normalize the counts of genes in each sample (using the BaseMean value to estimate the expression level), calculate fold changes, and perform differential expression analysis using a negative binomial (NB) test. Differential protein-coding genes were selected based on the fold change and significance of differential expression, with default criteria of q < 0.05 and fold change > 2. Finally, the transcriptome sequencing and analysis were conducted by OE Biotech (Shanghai, China).

### Statistical analysis

SPSS (version 26.0) statistical software was employed for statistical analyses. The data from at least three independent experiments are expressed as the mean ± standard error of the mean (SEM). Student’s t test or the Mann–Whitney U test was used to determine the significance of differences between two groups and Kruskal–Wallis was employed in more than 2 groups. For multiple comparisons, LSD correction was utilized for post hoc analyses. Kaplan–Meier survival curves along with the log-rank test were applied to survival analysis. P < 0.05 was considered statistically significant (*, p < 0.05, **, p < 0.01, ***, p < 0.001).

## Results

### SAE1 expression was upregulated in GC and was associated with Tumor Node Metastasis (TNM) staging, vascular invasion, and overall survival of gastric cancer patients

**Fig. 1:**
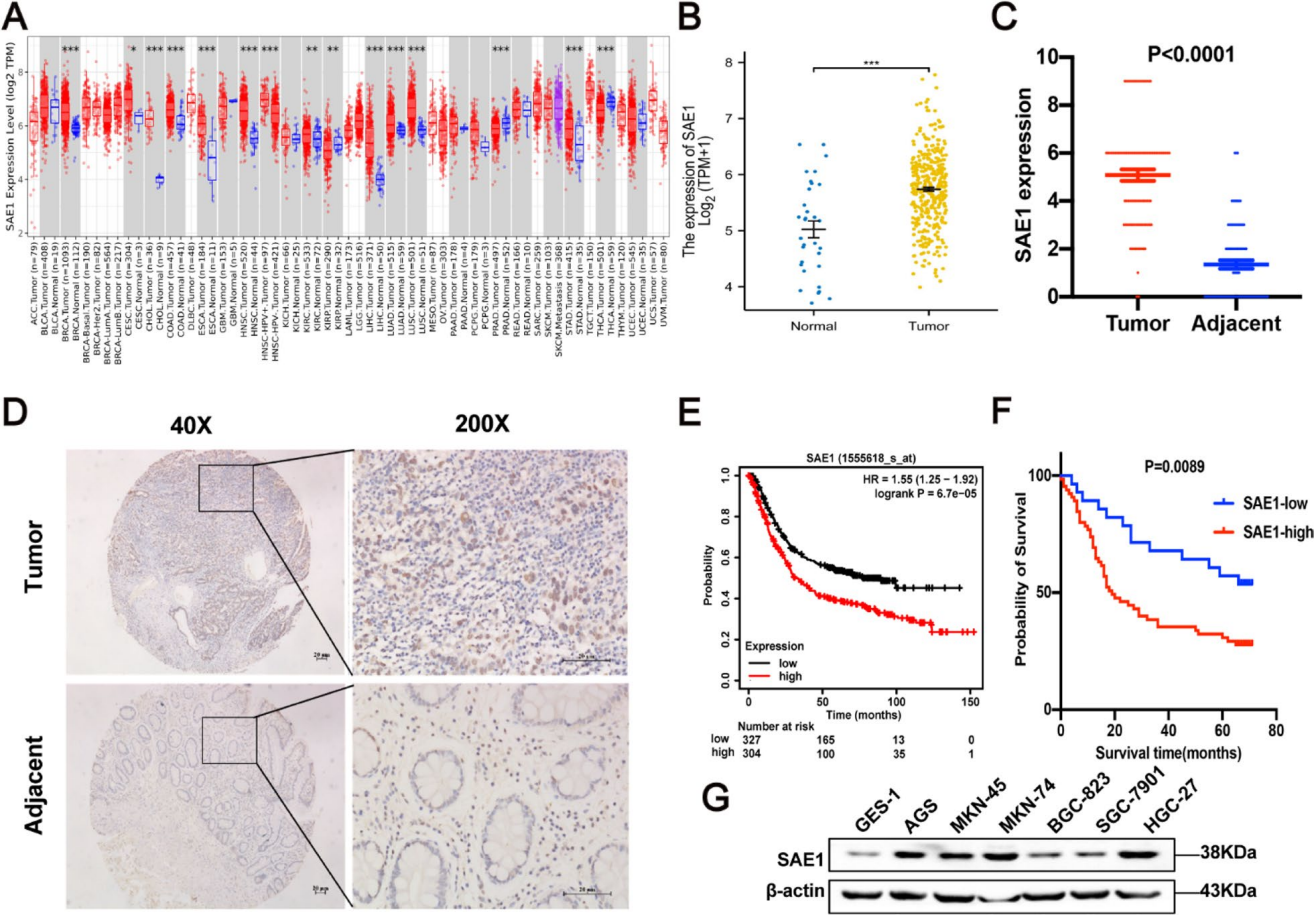
| SAE1 is overexpressed in gastric cancer (GC) and is associated with poor prognosis in GC patients. **A** The transcription levels of SAE1 in different tumor types from TCGA data analyzed in TIMER database. **B** Normalized expression value of SAE1 (log 2 transformation and Z correction) in GC tissues and adjacent tissues from the TCGA database. **C** The graph summarized immunohistochemical (IHC) scores of SAE1 expression in 93 pairs of GC and adjacent tissues from tissue microarray. **D** Representative images of IHC staining for SAE1 in human GC tissue microarray with 93 tumor tissues and paired adjacent tissues. (Magnification 40 × , 100 × , and 200 × ; bars = 20 μm). **E** The survival plot shows the prognosis in 304 GC patients with high SAE1 expression and 327 gastric cancer patients with low SAE1 expression from Kaplan–Meier Plotter database. **F** The survival plot shows the prognosis in 65 gastric cancer patients with high SAE1 expression and 28 gastric cancer patients with low SAE1 expression from GC tissue microarray. **G** SAE1 expression in several GC cell lines was analyses by western blot. The data is representative of 3 independent experiments

Initially we evaluated SAE1 mRNA expression levels in several human cancers by analyzing TCGA RNA-sequencing data in the TIMER database. SAE1 transcription level was remarkably higher in GC tissue compared with normal gastric tissue (Fig. 1A). We further used the TCGA database to confirm the higher SAE1 expression in tumor tissues compared with adjacent tissues (Fig. 1B), which was in line with IHC analysis from a GC tissue microarray (Fig. 1C, D). Both the survival analysis from the Kaplan–Meier Plotter database and GC tissue microarray demonstrated that higher SAE1 expression group indicated poorer prognosis than lower SAE1 expression group (Fig. 1E, F). Moreover, the clinicopathological characteristics of GC patients from the tissue microarray cohort were summarized in Table 1. Statistical analysis demonstrated that overexpressed SAE1 was markedly related to TNM staging, vascular invasion, and overall survival (Table 1).In addition, we detected SAE1 expression levels in several gastric cell lines. The SAE1 expression in several GC cell lines (AGS, MKN-45, MKN-74, BGC-823, SGC-7901, HGC-27) was higher than in gastric epithelial cells (GES-1) (Fig. 1G).

**Table 1:** Correlation between SAE1 protein expression and clinicopathologic characteristics of GC

Parameter	Total (n = 93)	SAE1 expression level		P Value
		High n = 65	Low n = 28	
Age (years)				
≥ 60	67	45	22	0.357
< 60	26	20	6	-
Gender				
Male	59	43	16	0.408
Female	34	22	12	
Tumor size				
≥ 5	60	42	18	0.976
< 5	33	23	10	
Pathological type				
Infiltrating	54	39	15	0.865
Elevated	7	5	2	
Ulcerative	30	20	10	
Grade				
I-II	40	32	8	0.065
III	53	33	20	
TNM stage				
I-II	35	20	15	0.029*
III-IV	56	44	12	
Vascular invasion				
No	65	41	24	0.029*
Yes	28	24	4	
Nerve invasion				
No	79	53	26	0.215
Yes	14	12	2	
Overall survival				
Survive	33	12	21	0.013*
Die	60	37	23	

^†^High and low expression was determined as total immunohistochemistry scores of 0–3 and 3–12 respectively

^*^P < 0.05 was identified as statistically significant

### SAE1 could promote proliferation, migration, invasion and metastasis in GC cells.

**Fig. 2:**
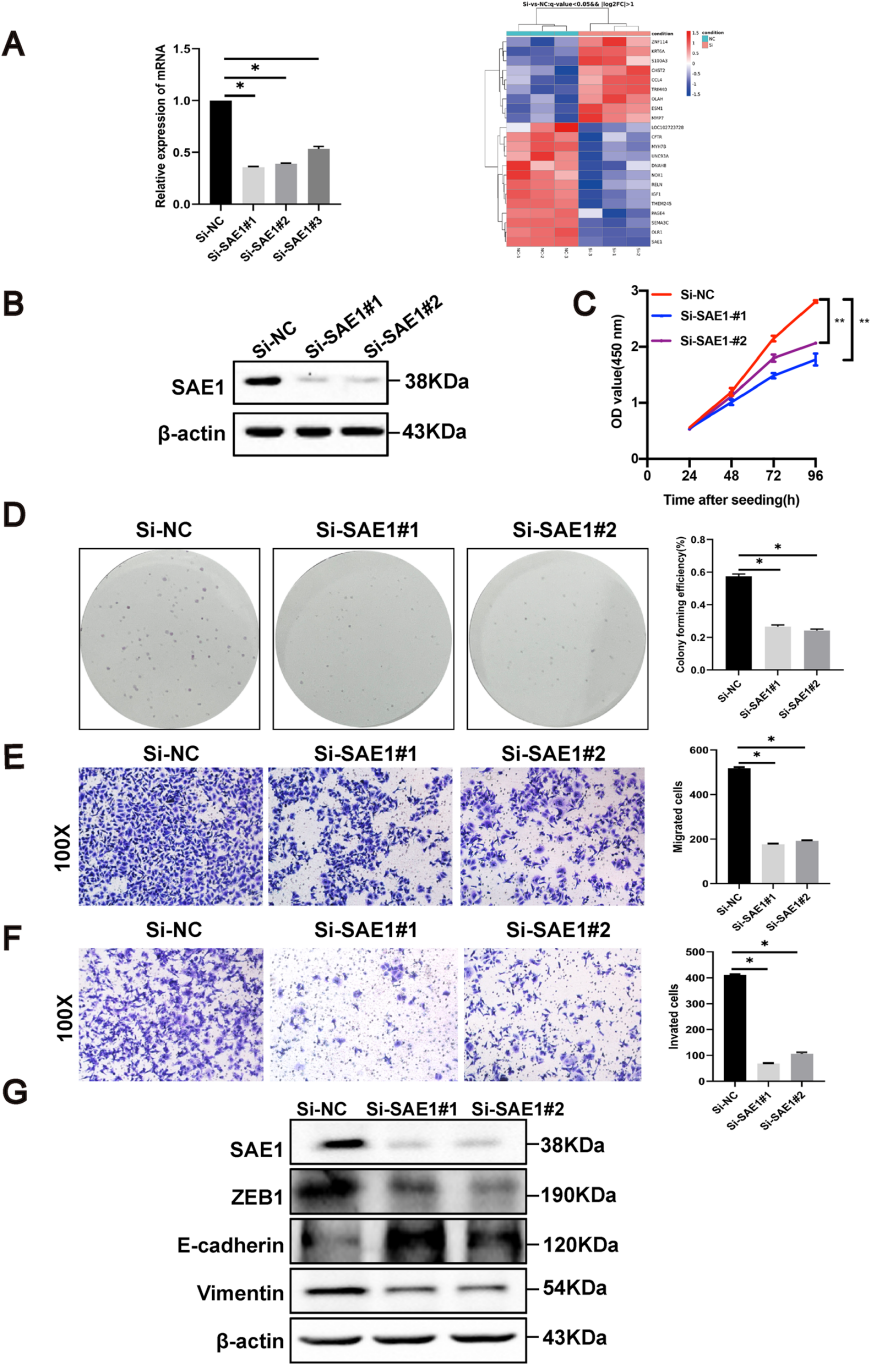
| SAE1 promotes proliferation, migration, and invasion of GC cells. **A** The heat map that showed the top 22 DEGs (differentially expressed genes) from analysis of RNA sequencing data in AGS cells with SAE1 knockdown and control group. **B** Western blot analysis of β-actin and SAE1 in AGS cells treated with negative control (Si-NC), SAE1 siRNA#1 (Si-SAE1#1), SAE1 siRNA#2 (Si-SAE1#2), and SAE1 siRNA#3 (Si-SAE1#3). The data is representative of 3 independent experiments. **C** CCK8 assays were conducted in AGS cells with Si-NC, Si-SAE1#1, and Si-SAE1#2, relative cell proliferation rate was represented as the OD value at a wavelength of 450 nm and was summarized in the statistical graph. The data is representative of 3 independent experiments. * p < 0.05, **p < 0.01. **D** Colony forming assays were conducted in AGS cells with Si-NC, Si-SAE1#1, and Si-SAE1#2, the colony forming efficiency was counted and summarized in the statistical graph. The data is representative of 3 independent experiments. * p < 0.05. **E** The migration assays were conducted in AGS cells with Si-NC, Si-SAE1#1, and Si-SAE1#2, positively stained cells were counted and summarized in the statistical graph. Original magnification, × 100. The data is representative of 3 independent experiments. * p < 0.05. **F** The invasion assays were conducted in AGS cells with Si-NC, Si-SAE1#1, and Si-SAE1#2, positively stained cells were counted and summarized in the statistical graph. Original magnification, × 100. The data is representative of 3 independent experiments. * p < 0.05. **G** Western blot analysis of β-actin, SAE1, Vimentin, E-cadherin, and ZEB1 in AGS cells with Si-NC, Si-SAE1#1, and Si-SAE1#2 treatment. The data is representative of 3 independent experiments

RNA sequencing of normal and SAE1 knockdown cells was performed to explore the influences of SAE1 in gastric carcinogenesis. The heat map showed the top differentially expressed genes (DEGs) (Fig. 2A). The primer sequences of SAE1 and GAPDH was listed in the Additional file 1: Table S3. GO analysis (Additional file 1: Fig S1) revealed that these DEGs was highly associated with inflammatory response, positive regulation of cell population proliferation, positive regulation of cell migration, etc. Particularly, IGF-1 (insulin like growth factor 1) was observed to be downregulated in cells after SAE1-silencing, while inflammatory cytokine, such as CCL4, was observed to be upregulated in the SAE1-silening cells.

To explore the impact of SAE1 expression on the proliferation, migration and invasion abilities, AGS cells were treated with SAE1 siRNAs to silence SAE1 expression. An obvious inhibition of SAE1 expression in AGS cells was performed (Fig. 2B) and was employed in subsequent functional experiments. CCK8 (Fig. 2C) and colony formation (Fig. 2D) assays were conducted to show that downregulation of SAE1 suppressed the proliferation of AGS cells. Inhibition of cell invasion and migration ability was also observed after SAE1 silencing using a Transwell assay (Fig. 2E, F).

In particular, the EMT phenotype was associated with cell migration and invasiveness. Our study showed that knockdown of SAE1 significantly upregulated the epithelial marker E-cadherin, and downregulated the mesenchymal marker Vimentin, and EMT-related transcription factor ZEB1 (Fig. 2G), suggesting that SAE1 could promote the EMT process in AGS cells.

### SAE1 was higher in *H. pylori*-infected gastric tissues than in *H. pylori*-uninfected gastric tissues.

**Fig. 3:**
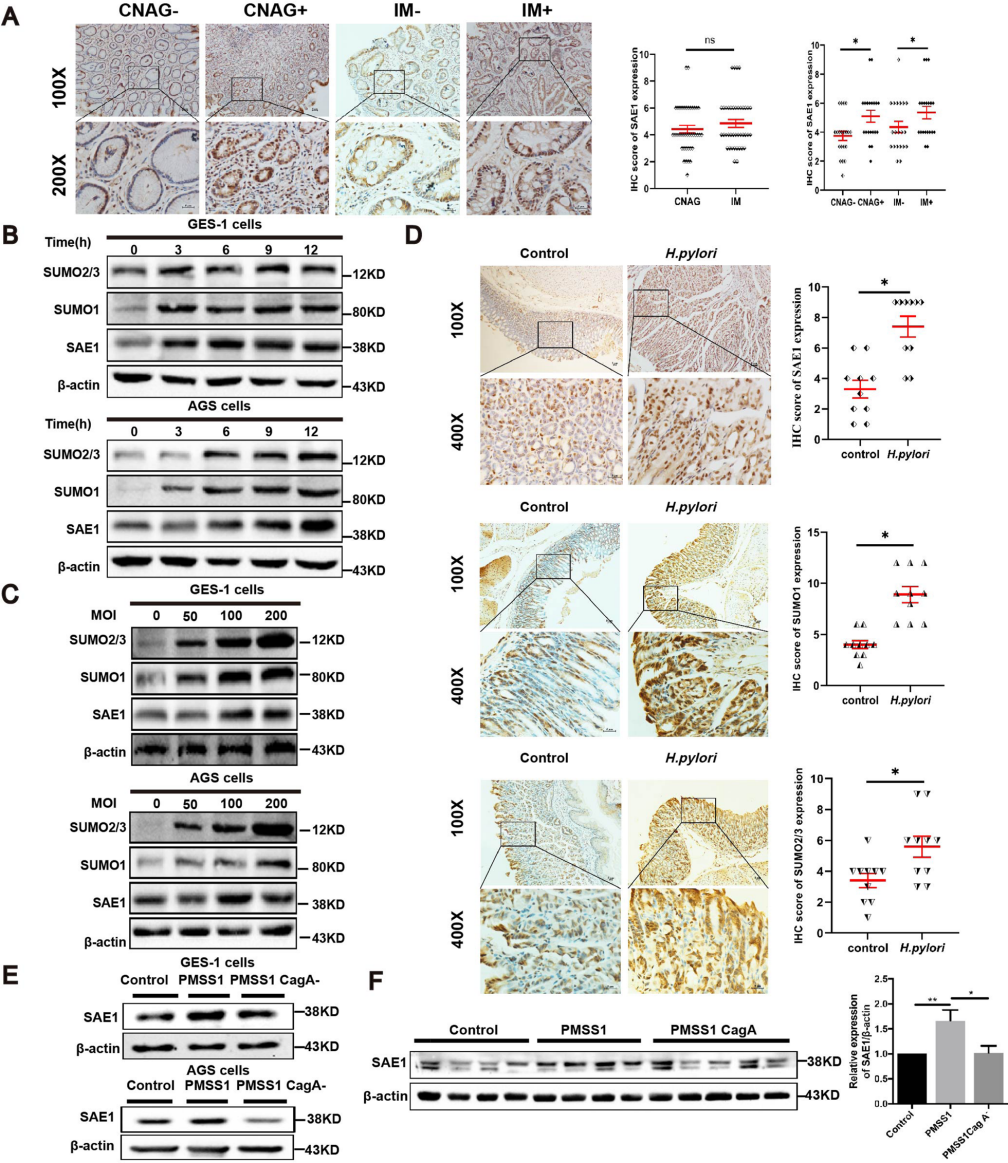
| SAE1 expression was higher in *H. pylori*-infected gastric tissues than in *H. pylori*-uninfected human gastric tissues. *H. pylori* upregulates SAE1 expression in GC cells and animal models dependent on CagA. SUMO1 and SUMO2/3 protein are also upregulated by *H. pylori* in cell and animal models. **A** Representative images of IHC staining for SAE1 in 80 human gastric tissues with *H. pylori*-negative chronic non-atrophic gastritis (CNAG) (n = 20), *H. pylori*-positive CNAG (n = 20), *H. pylori*-negative intestinal metaplasia (IM) (n = 20), *H. pylori*-positive IM (n = 20). (Magnification 100 × , and 400 × ; bars = 5 μm). The statistical graphs summarized IHC scores of SAE1 expression compared between human gastric tissues with CNAG and IM and IHC scores of SAE1 expression compared between *H. pylori*-negative and *H. pylori*-positive human gastric tissues with CNAG and IM. *p < 0.05, ns: no significance. **B** The protein levels of SAE1, SUMO1, and SUMO2/3 were detected in GES-1 and AGS cells cocultured with wild-type *H. pylori* strain PMSS1 with an MOI of 200 at different time points (0 h, 3 h, 6 h, 9 h, and 12 h). The data is representative of 3 independent experiments. **C** The protein levels of SAE1, SUMO1, and SUMO2/3 were detected in GES-1 and AGS cells cocultured with wild-type *H. pylori* strain PMSS1 at different MOI (0, 50, 100, and 200) for 12 h. The data is representative of 3 independent experiments. **D** Representative images of IHC staining for SAE1, SUMO1, and SUMO2/3 in Balb/c mice gastric tissues with control group (n = 10) and *H. pylori* strain SS1 group (n = 10). (Magnification 100 × , and 400 × ; bars = 5 μm). The statistical graphs summarized IHC scores of SAE1, SUMO1, and SUMO2/3 expression in Balb/c mice gastric tissues with control group, and *H. pylori* strain SS1 group. *p < 0.05. **E** Western blot analysis of SAE1 in GES-1 and AGS cells infected with wild-type *H. pylori* strain PMSS1 and its CagA^−^ mutant at an MOI of 200 for 12 h. The data is representative of 3 independent experiments. **F** SAE1 expression levels of C57BL\6 mice gastric mucosa infected with wild-type *H. pylori* strain PMSS1(n = 7) and its CagA^−^ mutant (n = 8) were analyses by western blot and were summarized in the statistical graph. Control group (n = 7). * p < 0.05, ** p < 0.01

*H. pylor*i was identified as a main carcinogenic factor of GC. To explore the relationship between SAE1 and *H. pylor*i, we conducted IHC staining to detect SAE1 expression in the gastric tissues of 80 patients with CNAG and IM. The distinct SAE1 expression between IM and CNAG tissues was not statistically significant (Fig. 3A). But the results suggested higher SAE1 expression in the *H. pylori*-infected gastric mucosa than in the *H. pylori*-uninfected mucosa (Fig. 3A). All these results suggested that higher SAE1 expression is highly associated with *H. pylori* infection.

### *H. pylori* upregulated SAE1, SUMO1, and SUMO2/3 expression in cell and animal infectious models

As SAE1 expression was higher in *H. pylori*-infected subjects, we further verified the effect of *H. pylori* on SAE1 expression in cell and mouse models. In cell models, GES-1 and AGS cells were cocultured with the *H. pylori* strain PMSS1 (Cag A^+^) for different periods of time or at different MOIs. Unexpectedly, SAE1 expression was upregulated at increasing periods of time during *H. pylori* infection. (Fig. 3B). SUMO1 and SUMO2/3 was observed to be upregulated by *H. pylori* as well **(**Fig. 3B, C). IHC analysis of animal models further confirmed these findings (Fig. 3D).

Cag A is a major virulence factor of *H. pylori*-induced gastric carcinogenesis. Cells were subsequently cocultured with wild type *H. pylori* strains PMSS1 and its Cag A^−^ isogenic mutants for 12 h to investigate the effect of Cag A in this process. The results showed that knockout of Cag A significantly attenuated SAE1 expression compared with the wild-type strain PMSS1 (Fig. 3E). Furtherly, C57/BL6 mice were infected with the PMSS1 strain and its Cag A^−^ isogenic mutant for 3 months before the protein of the mouse gastric mucosa was harvested. Western blot analysis showed the same results (Fig. 3F).

Generally, cell and animal models revealed that *H. pylori* infection upregulated SAE1 expression in a CagA-dependent manner. *H. pylori* can upregulate SUMO1 and SUMO2/3 expression as well.

### Suppression of SAE1 attenuated *H. pylori*-induced EMT in GC cells.

High SAE1 expression was significantly associated with TNM stage and vascular invasion according to the patient data of GC tissue microarray (Additional file 1: Table S1). Functional arrays also indicated that SAE1 promoted migration and invasion in GC cells (Fig. 2E, F). RNA sequencing analysis also showed that the DEGs between control group and SAE1 knockdown group was significantly involved in positive regulation of cell migration and cell proliferation (Additional file 1: Fig S1). Therefore, our study subsequently explored the relationship between SAE1, and *H. pylori*-induced EMT process.

**Fig. 4:**
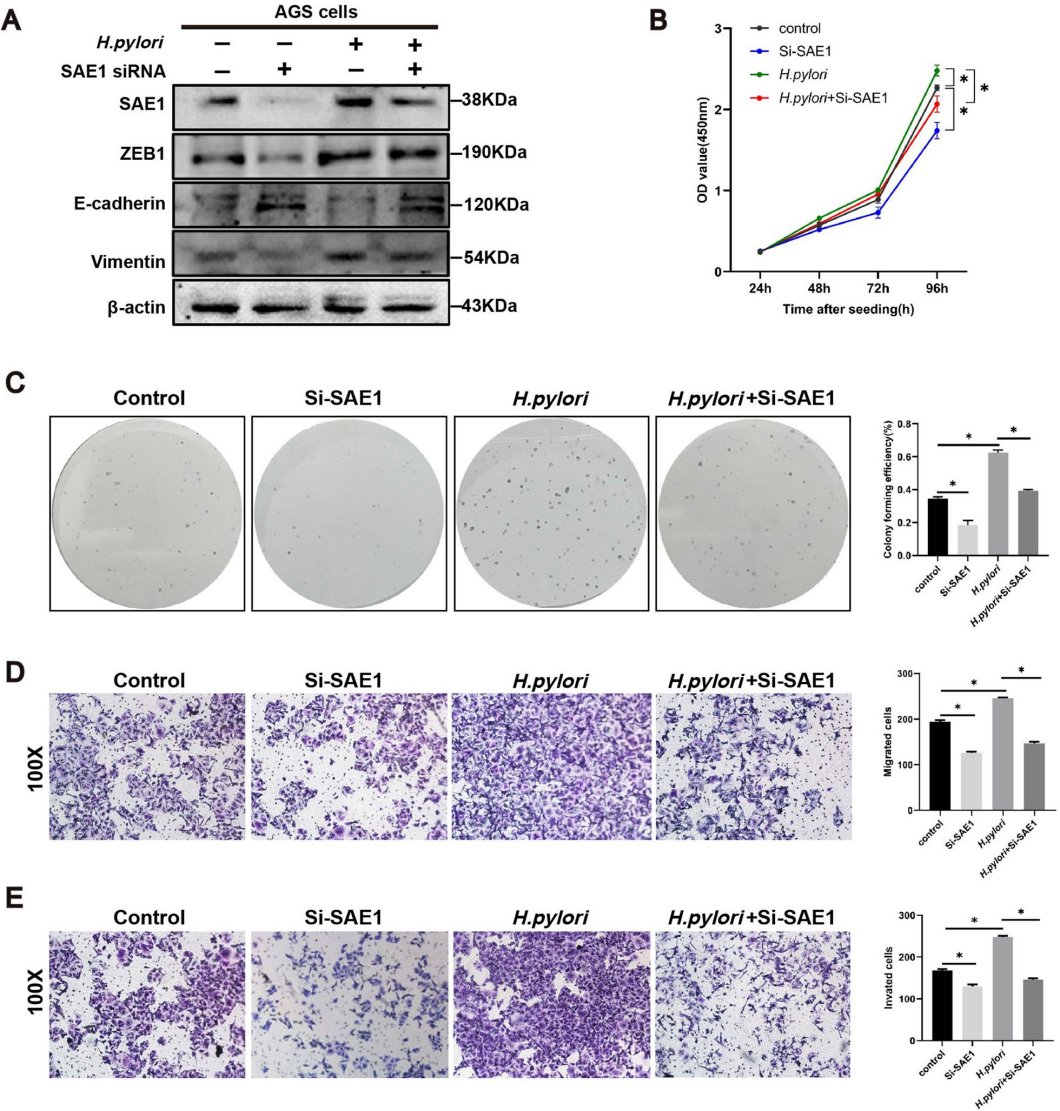
| Knockdown of SAE1 attenuates the epithelial–mesenchymal transition (EMT) program induced by *H. pylori* infection. **A** Western blot analysis of β-actin, SAE1, and markers of EMT process in AGS cells with control group, SAE1 siRNA transfection, infection of wild-type *H. pylori* strain PMSS1 (MOI = 200) for 12 h, and infection of wild-type *H. pylori* strain PMSS1 (MOI = 200) for 12 h after SAE1 siRNA transfection. The data is representative of 3 independent experiments. **B** CCK8 assays were conducted in AGS cells with control, Si-SAE1, *H. pylori*, and combination treatment of Si-SAE1 and *H. pylori* groups, relative cell proliferation rate was represented as the OD value at a wavelength of 450 nm and was summarized in the statistical graph. The data is representative of 3 independent experiments. *p < 0.05. **C** Colony forming assays were conducted in AGS cells with control, Si-SAE1, *H. pylori*, and combination treatment of Si-SAE1 and *H. pylori* groups, the colony forming efficiency was counted and summarized in the statistical graph. The data is representative of 3 independent experiments. *p < 0.05. **D** The migration assays were conducted in AGS cells with control, Si-SAE1, *H. pylori*, and combination treatment of Si-SAE1 and *H. pylori* groups, positively stained cells were counted and summarized in the statistical graph. The data is representative of 3 independent experiments. Original magnification, × 100. *p < 0.05. **E** The invasion assays were conducted in AGS cells with control, Si-SAE1, *H. pylori*, and combination treatment of Si-SAE1 and *H. pylori* groups, positively stained cells were counted and summarized in the statistical graph. The data is representative of 3 independent experiments. Original magnification, × 100. *p < 0.05

Our study showed that transient downregulation of SAE1 resulted in an increase in epithelial markers, and a decrease in mesenchymal markers. Combined treatment with siRNA transfection and *H. pylori* infection showed lower protein expression levels of Vimentin, and ZEB1, along with higher protein expression levels of E-cadherin, compared with the single *H. pylori*-infected group (Fig. 4A). Functional assays including CCK8 (Fig. 4B), colony formation (Fig. 4C), and transwell assays (Fig. 4D, E) indicated the same tendency. In general, SAE1 could facilitate the EMT phenotype and cell proliferation induced by *H. pylori*.

### ROS mediated the upregulation of SAE1, SUMO1, and SUMO2/3 by *H. pylori*

**Fig. 5:**
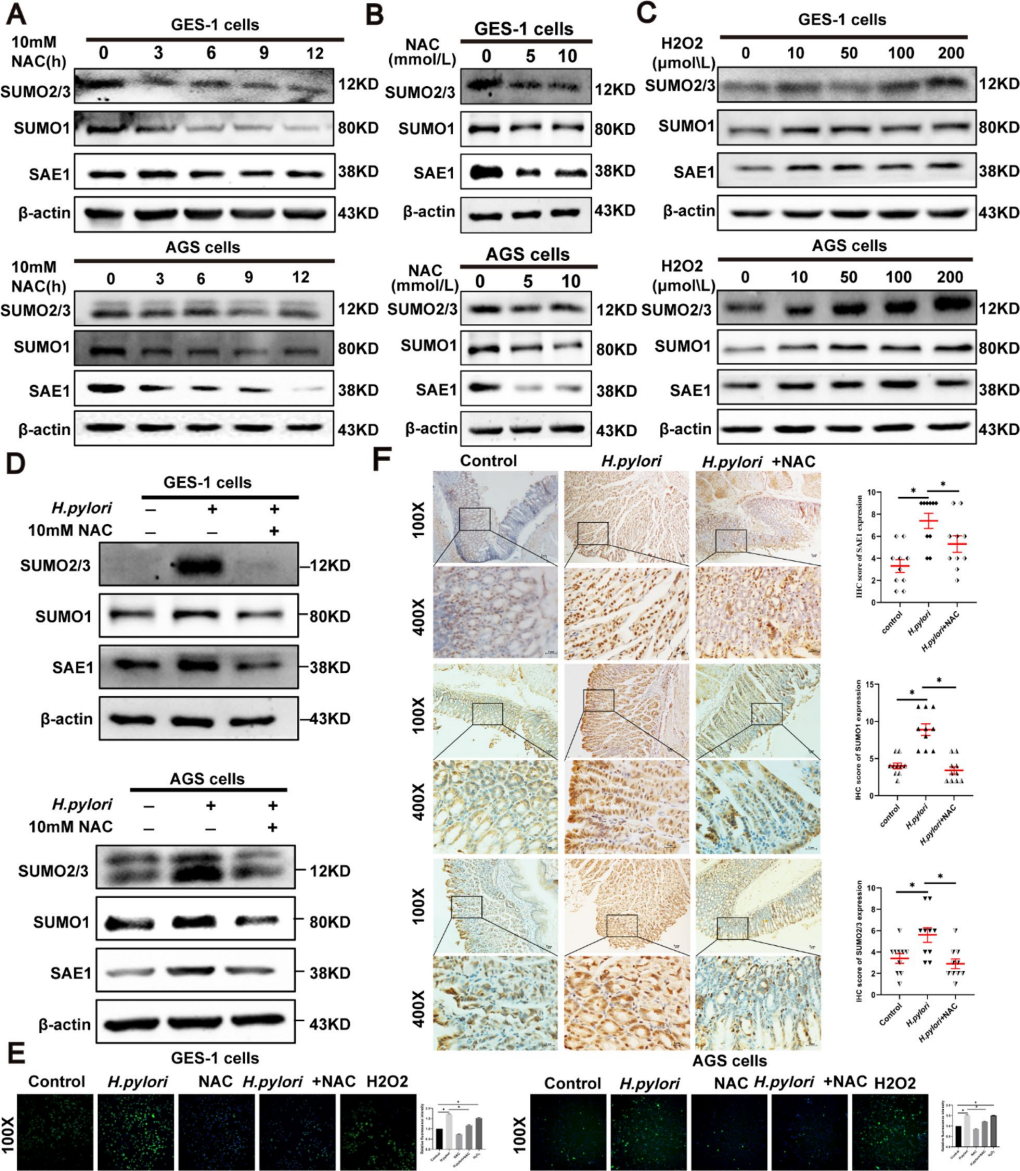
| ROS mediates the upregulation of SAE1, SUMO1, and SUMO2/3 by *H. pylori* in GC cells and animal models. **A** The protein levels of SAE1, SUMO1, and SUMO2/3 were detected in GES-1 and AGS cells treated with NAC (10 mmol/L) for different periods of time (0, 3, 6, 9, and 12 h). The data is representative of 3 independent experiments. **B** The protein levels of SAE1, SUMO1, and SUMO2/3 were detected in GES-1 and AGS cells treated with NAC in different concentrations (0, 5, and 10 mmol/L) for 12 h. The data is representative of 3 independent experiments. **C** The protein levels of SAE1, SUMO1, and SUMO2/3 were detected in GES-1 and AGS cells treated with H_2_O_2_ in different concentrations (0, 10, 50, 100, and 200 μmol/L) for 1 h. The data is representative of 3 independent experiments. **D** Western blot analysis of SAE1, SUMO1, and SUMO2/3 in GES-1 and AGS cells with control group, infection of wild-type *H. pylori* strain PMSS1 (MOI = 200) for 12 h, and combination treatment of wild-type *H. pylori* strain PMSS1 (MOI = 200) and NAC (10 mmol/L) for 12 h. The data is representative of 3 independent experiments. **E** The level of reactive oxygen species (ROS) in GES-1 and AGS cells was detected using H2DCFDA in control group, wild-type *H. pylori* strain PMSS1 (MOI = 200) infection for 12 h, NAC (10 mmol/L) treatment for 12 h, combination treatment of wild-type *H. pylori* strain PMSS1 (MOI = 200) infection and NAC (10 mmol/L) for 12 h, and H_2_O_2_ (200 μmol/L) for 1 h. Relative fluorescence intensity of ROS was summarized in the statistical graph. The data is representative of 3 independent experiments. *p < 0.05. **F** Representative images of IHC staining for SAE1, SUMO1, and SUMO2/3 in Balb/c mice gastric tissues with control group (n = 10), *H. pylori* strain SS1 group (n = 10), and combination of *H. pylori* strain SS1 and NAC group (n = 10). (Magnification 100 × , and 400 × ; bars = 5 μm). The IHC scores were summarized in the statistical graph. *p < 0.05

Previous studies have reported that *H. pylori*-induced ROS production has a great impact on gastric inflammation and carcinogenesis. ROS have a great impact on SUMOylation as well. Based on these findings, the ROS effect was subsequently investigated in the upregulation of SAE1. GES-1 and AGS cells were administered by NAC in a concentration gradient of 5 and 10 mmol/L and a time gradient of 0, 3, 6, 9, 12 h. SAE1, SUMO1 and SUMO2/3 expression were remarkably inhibited by NAC (Fig. 5A, B). When treated with H_2_O_2_ at different concentrations of 10, 50, 100, and 200 μmol/L. SAE1, SUMO1 and SUMO2/3 was upregulated in a concentration-dependent manner (Fig. 5C). Moreover, additional treatment of NAC inhibitor reversed the upregulation of SAE1 expression induced by *H. pylori* (Fig. 5D). Figure 5E shows the ROS production levels in different situations.

To further support these findings, Balb/c mice were treated with a single *H. pylori* SS1 strain, along with the combination treatment of the *H. pylori* SS1 strain and NAC inhibitor as previously described. IHC staining suggested a consistent results with cells (Fig. 5F). All these findings showed that ROS could mediate *H. pylori*-induced upregulation of SAE1.

**Fig. 6:**
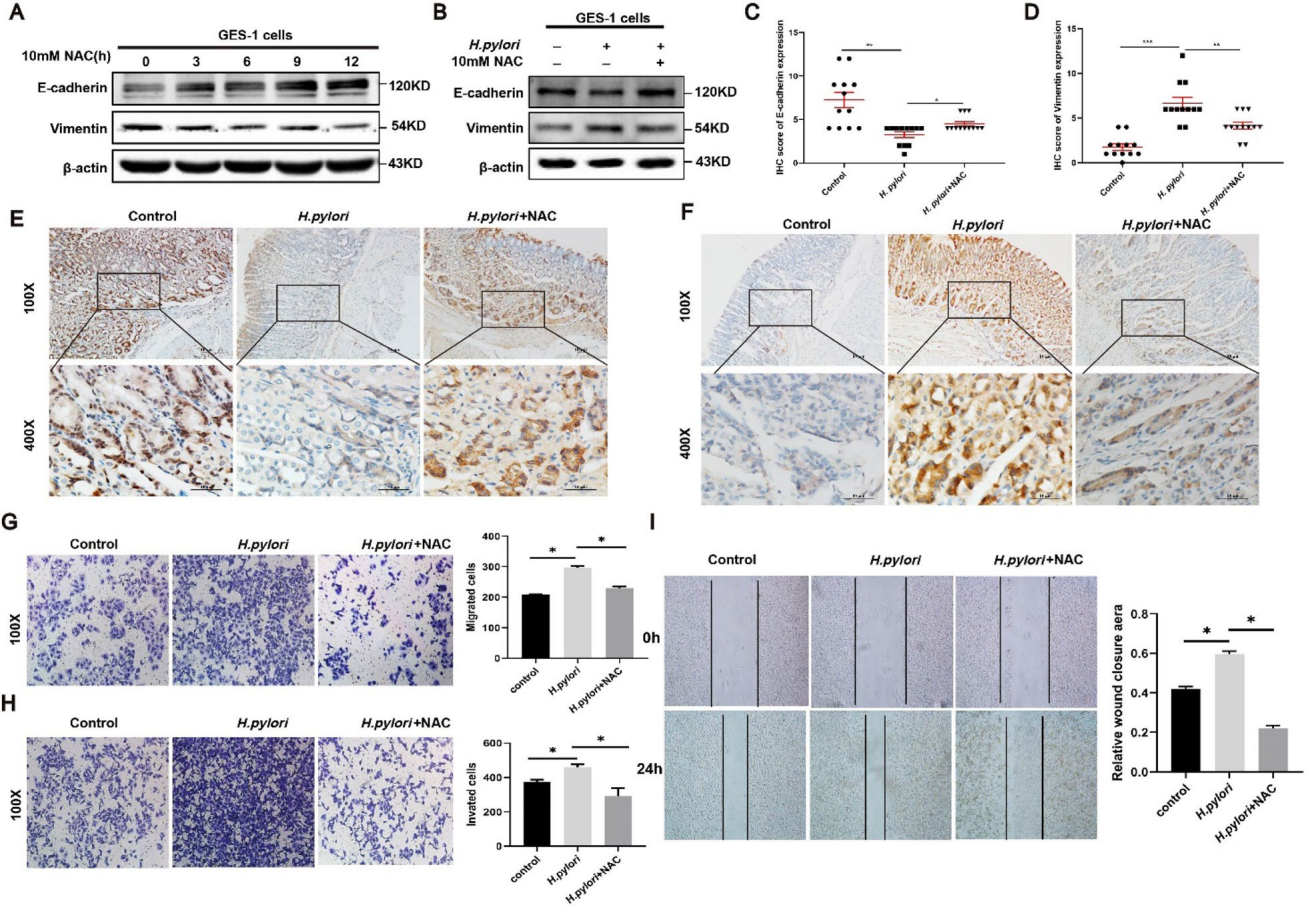
| ROS mediates cell proliferation and the EMT phenotype induced by *H. pylori* in GC cells. **A** The protein levels of β-actin, Vimentin, and E-cadherin were detected in AGS cells treated with NAC (10 mmol/L) for different periods of time (0 h, 3 h, 6 h, 9 h, and 12 h). The data is representative of 3 independent experiments. **B** Western blot analysis of β-actin, Vimentin, and E-cadherin in AGS cells with control group, infection of wild-type *H. pylori* strain PMSS1 (MOI = 200) for 12 h, and combination treatment of wild-type *H. pylori* strain PMSS1 (MOI = 200) and NAC (10 mmol/L) for 12 h. The data is representative of 3 independent experiments. **C**, **D** The IHC scores were evaluated and statistically compared for expression of E-cadherin and vimentin. **E**, **F** Representative images of IHC staining for E-cadherin and vimentin in Balb/c mice gastric tissues with control group (n = 10), *H. pylori* strain SS1 group (n = 10), and combination of *H. pylori* strain SS1 and NAC group (n = 10). (Magnification 100 × , and 400 × ; bars = 5 μm). **G** The migration assays were conducted in AGS cells with control, *H. pylori*, and combination treatment of NAC (10 mmol/L) and *H. pylori* groups for 12 h, positively stained cells were counted and summarized in the statistical graph. The data is representative of 3 independent experiments. (magnification × 100). **H** The invasion assays were conducted in AGS cells with control, *H. pylori*, and combination treatment of NAC (10 mmol/L) and *H. pylori* groups for 12 h, positively stained cells were counted and summarized in the statistical graph. The data is representative of 3 independent experiments. (magnification × 100). *p < 0.05. **I** The wound healing assays were conducted in AGS cells with control, *H. pylori*, and combination treatment of NAC (10 mmol/L) and *H. pylori* groups for 12 h. The scratch width was captured at 0 and 48 h after the scratch, the relative wound closure area was summarized in the statistical graph. The data is representative of 3 independent experiments. (magnification × 100). *p < 0.05, **p < 0.01, ***p < 0.001

We also observed the decreased the EMT-related markers in AGS cells treated by different time of NAC (10 mM) (Fig. 6A), inhibition of ROS in *H. pylori*-infected cells could reduce the E-cadherin protein while increased the Vimentin protein compared with single *H. pylori* groups (Fig. 6B). In addition, immunohistochemical analysis and quantitative image analysis revealed that gastric Vimentin expression was significantly increased and E-cadherin expression was decreased in *H. pylori*-infected mice compared with uninfected mice. However, gastric Vimentin expression decreased significantly and E-cadherin expression increased after NAC treatment (Fig. 6C–F). The following functional arrays including Transwell (Fig. 6G, H) and wound healing (Fig. 6I) also showed that elimination of ROS could reduce the EMT phenotype induced by *H. pylori*.

## Discussion

It is well-acknowledged that *H. pylori* infection is a main risk factor of GC. *H. pylori* infection contributes to chronic inflammation, stimulation of proliferation, oxidative stress that accumulates genetic mutations, and eventual gastric carcinogenesis [22]. SUMOylation-related enzymes have been documented to be correlated with many tumor types [10].

In our study, we detected the expression of SAE1 in a tissue microarray of GC and human gastric tissues of CNAG and IM. Overexpression of SAE1 was observed in GC and indicated poor prognosis in GC patients. Of the total CNAG and IM tissues, SAE1 expression was higher in the *H. pylori*-positive group than in the *H. pylori*-negative group. Subsequently, cell and animal models were employed to explore the relationships among SAE1, *H. pylori,* Cag A, and ROS. We found that Cag A is required for the upregulation of SAE1 induced by *H. pylori*. SUMO1 and SUMO2/3 was observed to be upregulated by *H. pylori* as well. ROS may mediate the upregulation of SAE1, SUMO1 and SUMO2/3. In addition, knockdown of SAE1 suppressed some malignant phenotypes, including cell proliferation, cell invasion, and cell migration. In particular, SAE1 promoted cell proliferation and the EMT program induced by *H. pylori*. RNA-sequencing analysis suggested that the IGF-1 was downregulated in the SAE1-silening cells, and Western blot analysis showed that knockdown of SAE1 significantly reduced IGF-1 (Additional file 1: Fig S2). Based on these data, ROS-mediated upregulation of SAE1 can promote carcinogenesis and progression of *H. pylori*-induced human GC in a Cag A-dependent manner.

SUMOylation is a reversible posttranslational modification that regulates crucial cellular functions and pathological processes. Recently, disturbed SUMOylation and deSUMOylation homeostasis has emerged as a fundamental route that may drive different steps of human tumorigenesis [23]. Our study suggested that SAE1 was highly expressed in GC and was associated with higher TNM stage, more vascular invasion, and poor overall survival. Suppression of SAE1 attenuated proliferation, migration, and invasion abilities of GC cells. Indeed, several SUMOylation-related components have been reported to be associated with GC. Multipronged OMIC analyses revealed that the SUMO E3 ligase Chromobox4 (CBX4) was overexpressed in GC and indicated poor prognosis [24]. In contrast, downregulation of PIAS3 [25] and PIAS1 [26] was observed in GC and was correlated with malignant phenotypes. The SUMOylated 5-methylcytosine (m5C) RNA methyltransferase NSUN2 could facilitate carcinogenic activity in GC [27]. SUMO-2/3-modified death domain-associated protein (DAXX) switches its opposing biological effects in GC by altering its subcellular localization [28]. Hence, complex interactions exist among SUMOylation-related components in gastric carcinogenesis.

Colonization of *H. pylori* strains is generally acknowledged as a risk factor for GC. The virulence factor Cag A can interact with many known cellular factors to manipulate intracellular signaling [6]. SUMOylation is regarded as an integral mechanism in bacterial infection and disease progression [29]. Bacterial pathogens intervene with host SUMOylation to ensure successful infection. Our study first shows the connections among SAE1, *H. pylori*, and Cag A. The wild *H. pylori* strain PMSS1 indeed upregulated SAE1 expression, whereas knockdown of Cag A weakened the effect. SUMO1 and SUMO2/3 expression was also observed to be upregulated by *H. pylori* infection. Moreover, Jaiswal et al. considered that histone-like DNA binding protein (Hup) of Helicobacter pylori, which is secreted by *H. pylori* and may contribute to immune-mediated gastric inflammation and epithelial damage, would bind to SUMO-proteins based on the presence of SIMs in Hup as well as charge complementarity between the Hup and SUMO-proteins [30]. All these findings established strong connections among *H. pylori*, Cag A and SUMOylation processes in multiple dimensions.

ROS are normal products of cellular metabolism. *H. pylori* has been identified to induce excessive ROS in gastric epithelial cells [3]. Accumulating evidence has revealed the regulation of SUMOylation by ROS, both in the context of normal redox signaling and severe oxidative stress [31]. SUMOylation also plays an essential role in the regulation of ROS production and clearance to maintain a balance of ROS homeostasis [9]. Our results indicated that ROS mediated the upregulation of SAE1 by *H. pylori*. Additional NAC inhibitor treatment inhibited *H. pylori*-induced expression of SAE1. Previous studies indicated that oxidative stress induced by H_2_O_2_ indeed increased the SUMOylation of NF-κB essential modulator (NEMO) (2 mM, 40 min) [32, 33] and ERM (100 mM, 20 min) [34]. Our findings showed that oxidative stress also exerts impact on SUMOylation-related E1-activating enzyme.

The EMT program is defined as a process by which cells lose their junctions and adhesion structures to achieve motility, invasion, and metastasis [18]. An increasing number of findings support that *H. pylori* infection leads to the progression of EMT program in GC dependent on its virulence factor Cag A [35, 36]. According to our findings, *H. pylori* infection contributed to increased Vimentin, and ZEB1 expression along with decreased E-cadherin expression. However, treatment with SAE1 silencing significantly reversed the *H. pylori*-induced alteration of these EMT-related hallmarks and decreased the invasion and migration ability of GC cells. Particularly, our RNA sequencing analysis reveals that silencing of SAE1 has an obvious impact on the mRNA expression of IGF-1. IGF-1, which belongs to IGF system, was reported to induce the EMT phenotype and promote cancer proliferation in breast, lung, and gastric Cancers [37]. Downregulation of SAE1 may disrupt the SUMOylation and deSUMOylation homeostasis, thus affecting the EMT program via exerting an influence on IGF-1 expression.

## Conclusion

Generally, our findings suggests that SAE1 is overexpressed in GC and indicates poor prognosis. Both cell and animal models suggest that *H. pylori* infection upregulates SAE1, SUMO1 and SUMO2/3 expression. ROS can mediate this process. Knockdown of SAE1 can suppress cell proliferation and EMT phenotype induced by *H. pylori*. Our findings reveal considerable role of SAE1 in the *H. pylori*-associated GC, suggesting that targeting SAE1, the SUMOylation-related component, might be a promising strategy for GC therapy.

### Supplementary Information

**Additional file 1: Table S1.** The Sequences of siRNAs for Target SAE1. **Table S2.** Antibodies Used in western Blot, and immunohistochemistry Staining. **Table S3.** The primer sequences of SAE1 and GAPDH. **Figure S1.** The top 22 GO terms from analysis of DEGs (differentially expressed genes) in RNA sequencing data btween SAE1 knockdown and control group cells. **Figure S2.** Western blot analysis of β-actin, SAE1 and IGF-1 in AGS cells with Si-NC, Si-SAE1#1, and Si-SAE1#2 treatment. **Figure S3.** The top KEGG terms from analysis of DEGs (differentially expressed genes) in RNA sequencing data between SAE1 knockdown and control group cells.

## Data Availability

The original contributions presented in the study are included in the article/supplementary material. Further inquiries can be directed to the corresponding authors. For researchers who are interested in accessing the code or dataset used in this study, please feel free to contact the corresponding author directly. We would be happy to provide any additional information or assistance required.
