# Supplementary material for: ROS-mediated up-regulation of SAE1 by Helicobacter pylori promotes human gastric tumor genesis and progression
Source: J Transl Med. 2024 Feb 13;22:148. doi: 10.1186/s12967-024-04913-5 (PMC10863176; doi:10.1186/s12967-024-04913-5)
Supplement: Supplementary file 1 — Additional file 1: Table S1. The Sequences of siRNAs for Target SAE1. Table S2. Antibodies Used in western Blot, and immunohistochemistry Staining. Table S3. The primer sequences of SAE1 and GAPDH. Figure S1. The top 22 GO terms from analysis of DEGs (differentially expressed genes) in RNA sequencing data btween SAE1 knockdown and control group cells. Figure S2. Western blot analysis of β-actin, SAE1 and IGF-1 in AGS cells with Si-NC, Si-SAE1#1, and Si-SAE1#2 treatment. Figure S3. The top KEGG terms from analysis of DEGs (differentially expressed genes) in RNA sequencing data between SAE1 knockdown and control group cells. [file 12967_2024_4913_MOESM1_ESM.docx]

**Additional file**

| **Additional file Table S1. The Sequences of siRNAs for Target SAE1** | | | |
| --- | --- | --- | --- |
| **Item** | **Item name** | **sense（5'-3'）** | **antisense（5'-3'）** |
| **SAE1#1** | **SAE1-Homo-213** | **GCUUCUUGUCGGCUUGAAATT** | **UUUCAAGCCGACAAGAAGCTT** |
| **SAE1#2** | **SAE1-Homo-726** | **GGUGGUCUUCUGCCCUGUUTT** | **AACAGGGCAGAAGACCACCTT** |
| **SAE1#3** | **SAE1-Homo-1058** | **GGGACCCUCCUCACAACAATT** | **UUGUUGUGAGGAGGGUCCCTT** |

| **Additional file Table S2. Antibodies Used in western Blot, and immunohistochemistry Staining** | | | |
| --- | --- | --- | --- |
| **Antibody** | **Catalog NO.** | **Company** | **Country** |
| **anti-β-actin** | **M176178** | **TransGen Biotech** | **China** |
| **anti-SAE1** | **10229-1-AP** | **Proteintech** | **USA** |
| **anti-SUMO1** | **ab32058** | **Abcam** | **UK** |
| **anti-SUMO2/3** | **11251-1-AP** | **Proteintech** | **USA** |
| **anti-Vimentin** | **10366-1-AP** | **Proteintech** | **USA** |
| **anti-E-cadherin** | **20874-1-AP** | **Proteintech** | **USA** |
| **anti-ZEB1** | **20874-1-AP** | **Proteintech** | **USA** |
| **Goat anti-Rabbit HRP IgG** | **A0216** | **Beyotime** | **China** |
| **Goat anti-Mouse HRP IgG** | **A0428** | **Beyotime** | **China** |

| **Additional file Table S3. The primer sequences of SAE1 and GAPDH** | | |
| --- | --- | --- |
| **Item** | **forward primers** | **reverse primers** |
| **GAPDH** | **5′-TGGAAGGACTCATGACCACA-3′** | **5′-TTCAGCTCAGGGATGACC TT-3′** |
| **SAE1** | **5′-TGGAGCAGTGAGAAAGCAAAG-3′** | **5′-GGAAGCAGGTCAGGACTAATAC-3′** |

**Additional file Figure S1**

**
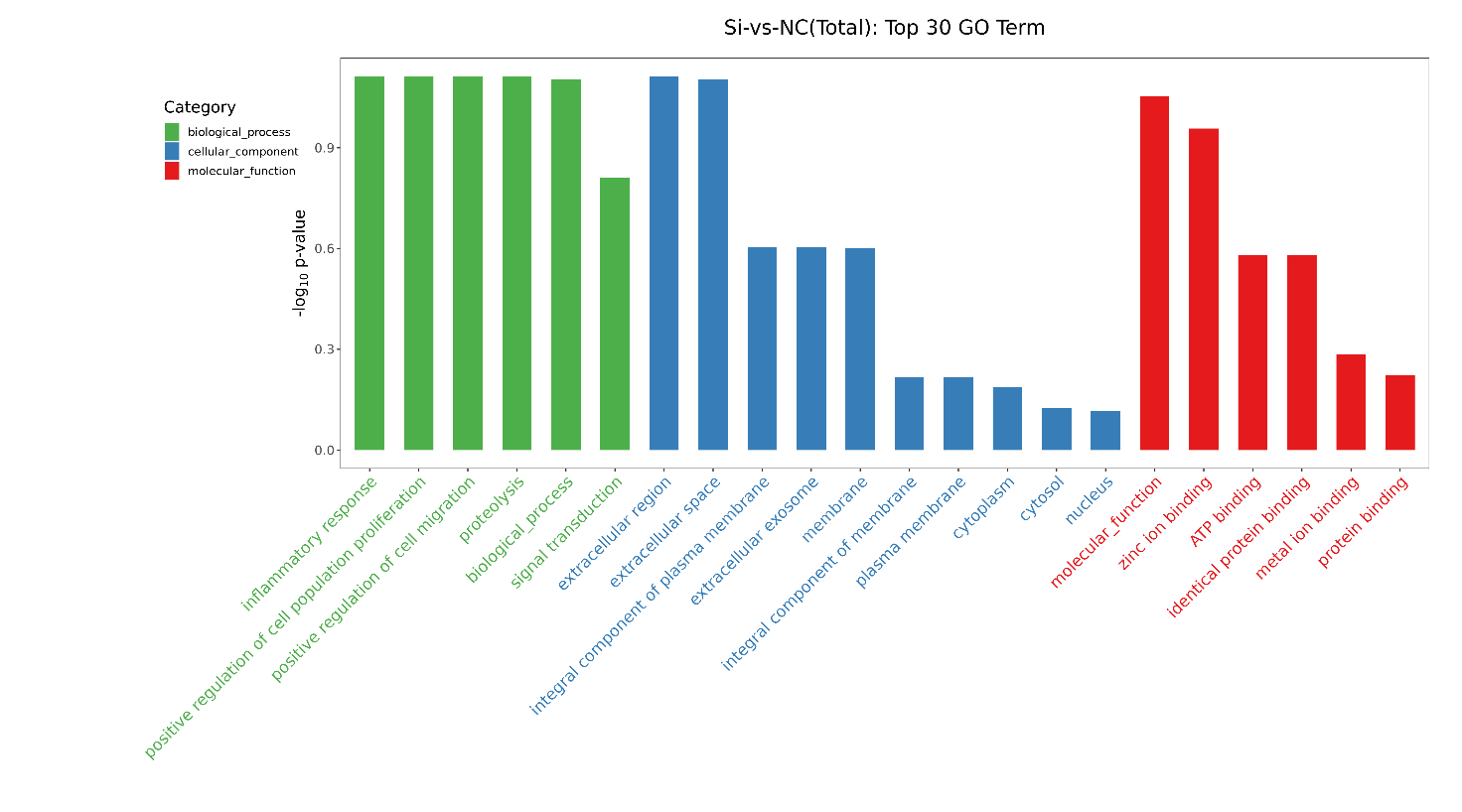
**

Figure S1. The top 22 GO terms from analysis of DEGs (differentially expressed genes) in RNA sequencing data btween SAE1 knockdown and control group cells.

**Additional file Figure S2**

**
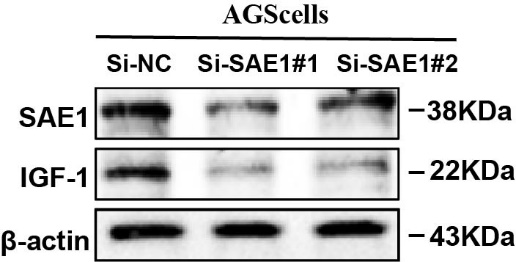
**

Figure S2. Western blot analysis of β-actin, SAE1 and IGF-1 in AGS cells with Si-NC, Si-SAE1#1, and Si-SAE1#2 treatment.

**Additional file Figure S3**

**
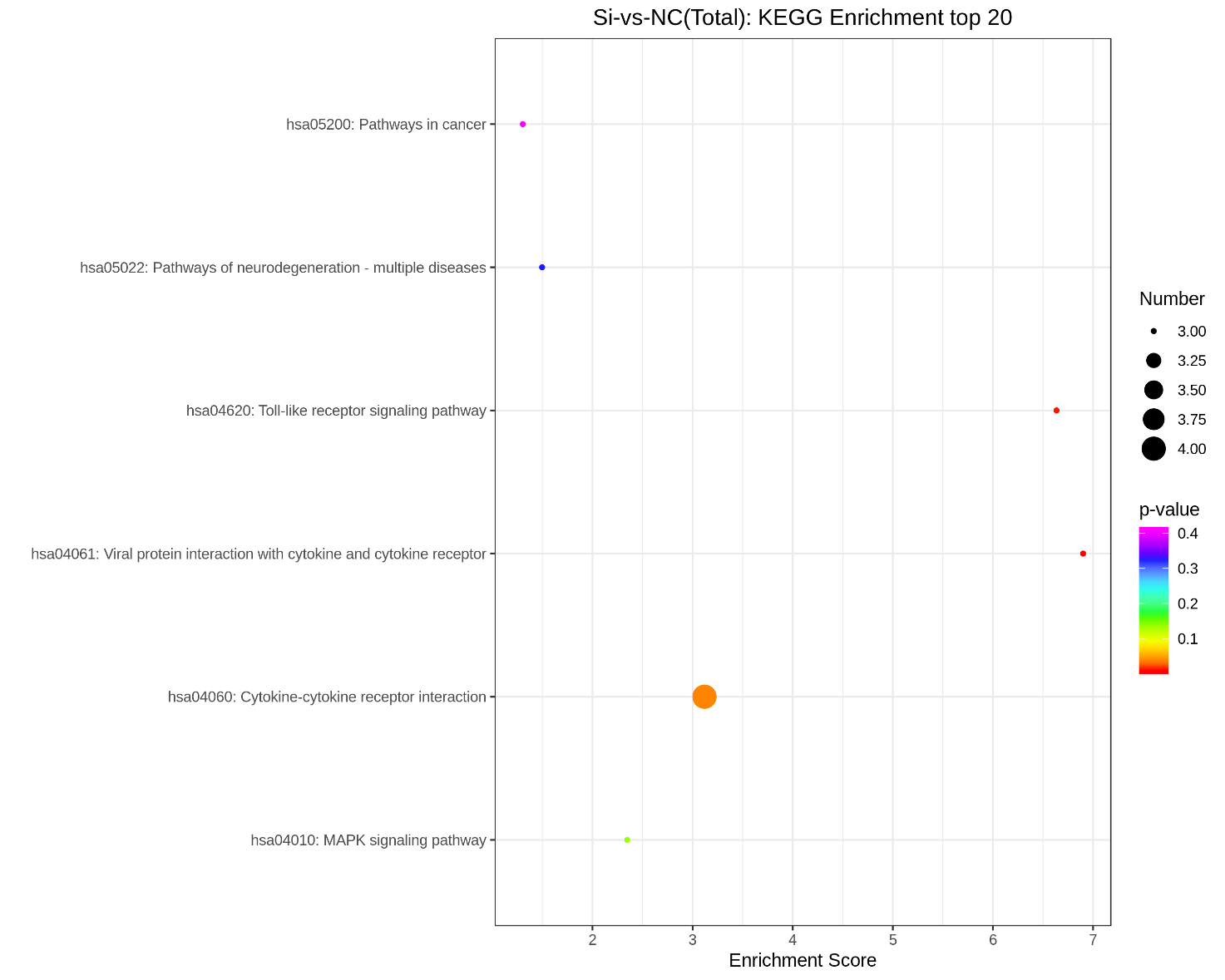
**

Figure S3. The top KEGG terms from analysis of DEGs (differentially expressed genes) in RNA sequencing data between SAE1 knockdown and control group cells.
